# Geometry to Build Models, Models to Visualize Geometry

**DOI:** 10.1007/s40751-020-00080-6

**Published:** 2020-09-21

**Authors:** Caterina Cumino, Martino Pavignano, Maria L. Spreafico, Ursula Zich

**Affiliations:** 1 Department of Mathematical Sciences “G. L. Lagrange” (DISMA), Politecnico di Torino, corso Duca degli Abruzzi 24, 10129 Torino, Italy; 2 Department of Architecture and Design (DAD), Politecnico di Torino, viale Pier Andrea Mattioli 39, 10125 Torino, Italy

**Keywords:** Calculus, Architectural drawing, Developable surfaces, Representation issues, Physical models, Undergraduate architecture

## Abstract

In the seventeenth century, Guarino Guarini, mathematician and architect, affirmed that architecture, a discipline that primarily deals with measures, relies on geometry: therefore, the architect needs to know at least its basic principles. On behalf of Guarini’s words, we designed a set of interdisciplinary teaching experiences, between mathematics (via a calculus course) and drawing (via our Architectural Drawing and Survey Laboratory courses) that we proposed to first-year under graduate students studying for an Architecture degree. The tasks concern mathematical and representational issues about vaulted roofing systems and are based on the use of physical models in conjunction with digital tools, in order to make the cognitive geometric process more effective, thus following a consolidated tradition of both disciplines.

## Introduction

In this article, we describe and discuss a set of teaching experiences, presented to undergraduate students (in an Architecture degree, first year), that is a result of an interdisciplinary research project developed between mathematics and drawing. The students’ activity was focused on investigating mathematical and representational issues related to vaulted roofing systems, concerning vaults generated by cylinders and intersections between them. Our aim is to enhance students’ geometrical comprehension of architectural shapes, stimulating their spatial visualization ability, in the sense of Leopold (2015) and Nagy-Kondor (2007, 2010).

In fact, in recent years, many scholars in different research fields have pointed out an increasing difficulty in geometric and spatial visualization by students starting their undergraduate studies (Ragni and Knauff 2013; Jones and Tzekaki 2016; Kovačević 2017), especially in places where geometry is still being reduced in favour of numeracy skills (Kovačević 2017). This trend is not so new, at least in the Italian context (Mammana and Villani 1998).In the Italian academic panorama, this is even truer if we analyse the situation of architectural studies, where most of the students do not perceive connections between mathematics and other disciplines (Cumino et al. 2019),^1^ for they have a tendency to compartmentalize knowledge (Hiebert and Lefevre 1986), especially within mathematics learning and in applying mathematical topics to other disciplines, or vice versa. The ability to identify and represent the same mathematical concept through different representations and to select and use different solution strategies is considered as a fundamental process leading to mathematical understanding and successful problem solving (Duval 2006).

Our first-year Architecture undergraduate students take a traditional calculus course during the first semester, including topics of linear algebra, analytic geometry, differential and integral calculus.^2^ Context and temporal position forces teachers to use a variety of cognitive artefacts, both tangible and virtual, to obviate for some a lack of knowledge due to the heterogeneous high-school provenance. The partial simultaneity of calculus and the Architectural Drawing and Survey Laboratory (hereafter, ADSLab) courses led, over the years, to experimentation with students’ interdisciplinary activity on vaulted surfaces (Cumino et al. 2017a), in which we try explicitly to bring out connections between architects’ main language, drawing and representation (De Fusco 2010; Cardone 2016) and mathematical aspects of architectural shapes (in the spirit of descriptive geometry) (Salvadori and Levy 1967; Stachel 2003, 2015).

### Geometry between Architecture and Mathematics^3^

Inter connections through geometry between architecture and mathematics can be dated back at least to ancient Egypt and have been investigated in classical Greece (Smith 2004). Following this path, in the first century BC, Marcus Vitruvius Pollio wrote that architects should have competences in many fields, including drawing and mathematics (Vitruvius Pollio 1960, pp. 315–316). After many centuries, one of the most important mathematicians and architects of the Italian baroque, Guarino Guarini (1737), stated that, “being a profession which uses measures in each of its operations, Architecture relies on Geometry, thus he [the architect] needs to know at least its basic principles” (p. 3).^4^ Moreover, geometry, within mathematics – according to the architect Ludovico Quaroni (1977) – deals with theoretical space, while architecture, as result of the sum between art and technique (basically recalling the Greek term composed from *ἀρχή*-*archē* and *τέχνη-téchne *combined) deals with tangible space. Thus, it is clear that geometry, both in its architectural and mathematical contexts, can be a powerful tool to share and improve students’ knowledge of architectural problems (Williams and Ostwald 2017).

### Enhancing Mathematical Cognition in Architecture Curriculum

The cultural environment to which we belong is characterized by a high grade of interdisciplinarity, as it is one of the last outcomes/legacies of the *École Polytechnique*. It gave us spaces and opportunities to tackle the problem highlightedinthe next section, working in two courses of the Architecture degree (calculus, ADSLab; academic year 2018–2019). In this context, we developed tangible interactive aids in teaching geometry, between mathematics and representation, and we investigated their cultural values in an architect’s training path, with specific attention to the experience in the ADSLab.

We are aware that, nowadays, teaching aids are increasingly dematerialized: however, it is our conviction that virtual models risk being used as black boxes.At university, students can solve more and more complex problems using computer software, while there is still a lack of basic mathematical understanding, as remarked by Stachel (2015) about descriptive geometry education.

To make the cognitive geometric process more effective, in our lessons we have introduced, in conjunction with digital tools, the use of scale physical models as interactive teaching media, which are at the same time both tangible representations and tools to verify design assumptions. In this way, we follow a tradition that sees the importance of using physical models both in architecture and in mathematics.

## The Use of Physical Models as Interactive Teaching Media to Enhance Geometric Spatial Knowledge^5^

In this section, we introduce theoretical foundations of physical models in our reference disciplines, architecture and mathematics. We have to frame our research project inside a wider panorama, one that has its roots from a fairly countless number of years ago. Both architectural and mathematical physical models usually proposed both a material, scaled representation of mental models (Klein 1894; Friedman and Krausse 2016) and a tangible description of built artefacts (Gay 2015). Only in recent years, especially in architecture, has the role of the physical model been analysed and critically evaluated by scholars.

### Physical Models in Architecture^6^

The use of scale/physical models in architecture has always been an important point of intersection between theory and practice for technical professions in the field of building sciences. Historically, it draws its origins from the material representations of human beings and buildings used for religious ceremonies and magic rituals. These models were reductions of everyday life scenes, performing different celebrative, votive or ludic roles (Scolari 2005; Barlozzini 2013; Smith 2004).

In the architectural context, the scale/physical model’s basic function has always been that of *exemplum* for detail realization or of allowing architects and engineers, “to predict the future by interpreting signs and omens” (Smith 2004, p. 2), But it also has been a good tool to help develop an understanding of shapes/designs/ideas, while acting as “writing for illiterates” (Scolari 2005, p. 132). In his treatise *On the art of building in ten books*, the renaissance architect and humanist Leon Battista Alberti described physical/scale models as instruments with which to think about architectural design, to test projects (in terms of both technical and quantitative evaluations), to interact easily with craftsmen (Smith 2004, pp. 25–30). Alberti’s example is useful to document the main uses of models in architecture prior to the computer revolution.

In fact, nowadays, the general statute of the term *model* has become extremely complex, no longer attributable to the simple definition of a *datum* to be reproduced or copied (Ugo 2008, p. 21), nor to a tangible or digital artefact. In other words, the model itself is the result of a complex process of critical analysis and synthesis of the architectural project or of the built space. Thus, it is not a simple Greek *παράδειγ*μα*-parádeigma *(specimen or example produced in order to study specific and detailed 3D architectural elements) nor an instrument to determine the architectural elements that could be interpreted and changed (Smith 2004, pp. 10–11).

### Physical Models in Mathematics^7^

Mathematics deals with concepts, ideas, objects, which are not directly accessible, unlike physical objects. Hence, there is a need for tools and methods to describe and indicate them: in other words, mathematical thinking must make use of representations (Duval 1999). These can be a set of symbols, formulas or visualizations through images external to the mind (such as diagrams, drawings, physical and virtual models, etc.) or visualizations through mental images. Visualization is widely recognized to play an important role in doing, teaching and learning mathematics, see e.g. Nelsen (2016), even if the term is used in literature with a large variety of meanings: we just mention the description of visualization (Nemirovsky and Noble 1997) as the means of travelling between external representations and the learner’s mind; for an overview on the subject, see the recent research review by Jones and Tzekaki (2016).

Due to the nature of mathematical objects, as asserted by Duval (2017), “The association between the representations and the object itself, the words and the designated things, a work and its model etc., appear as the fundamental cognitive process to ‘make sense’ and to verify, and hence, acquire new knowledge” (pp. 27–28). Studies on using material tools in mathematics education generally indicate that physical representations play an important role at all educational levels, not only in learning processes of mathematical ideas, but also in problem-solving settings, although they need caution to ensure understanding and meaningful learning (Friedman and Krausse 2016). The visual sense then becomes a central component of the learning experience (Sarama and Clements 2016).

Physical models have a long tradition in mathematics. At the end of the eighteenth century, Gaspard Monge realized the first models of ruled surfaces in metal and silk threads, for his lectures at the *École Polytechnique* in Paris, and some students also produced specimens with dynamic properties (Paessler and Lordick 2019). In the second half of nineteenth century, progress in the study of geometry led many mathematicians to build models of algebraic surfaces and curves (see Giacardi 2015). They had interactions both with research, to provide an effective mental image of research objects (Klein 1894), and with teaching at the university level, not only in mathematics, but also in other disciplines, such as civil engineering and architecture. In the following decades, the prevalence of a more abstract point of view in mathematical research decreased interest in model production. However, towards the middle of the last century, some scholars – such as Emma Castelnuovo (1965) – already realized the great importance of the so-called ‘intuitive teaching by images’, in contrast to the traditional verbal and abstract teaching, and they advocated a multimodal learning of mathematics, developed (especially for geometry) through the use of concrete materials and movement.

In the final decades of the twentieth century, developments in information technology made it possible to construct a new typology of models that allowed not only visualizing static images of abstract mathematical objects, but also modifying objects and observing them from different points of view, with undoubted advantages both for research and for teaching. In particular, dynamic geometry software programs revealed their potential in the development of visualization skills, although some scholars warn against their exclusive use (see, for example, Maracci 2018), at the expense of bodily and motor experience in learning dynamics.

Folded-paper models deserve a separate place in the history of models. At the end of the nineteenth century, folding was considered a mathematical tool and a way to express essential features of a geometric form (Wiener and Treutlein 1912; Friedman and Krausse 2016). Folded models were tangible like other models, with the added value that the transformation involved in their production (from a 2D sheet of paper to a 3D shape) had a precise mathematical meaning and gave the model dynamic properties.

## Models to Visualize Geometry of Vaults and Roofing Systems in Education^8^

The ADSLab is part of the first-year educational path of architecture at the Politecnico di Torino (Italy) and aims to build the scientific foundations (theoretical and applicative) of language and codes of drawing. The laboratory consists of three main areas (fundamentals and applications of descriptive geometry, digital drawing and modelling, drawing and surveying) which use various tools of representation to describe, for example, roof surfaces. There are many variations of the models for describing them (Spallone and Vitali 2017).The following is a description of what happens in our ADSLab: physical and virtual models are used in the observation and recognition of basic geometries, for investigation, representation, measure, calculus and design.

Making geometry tangible educates spatial vision, interpretation of architectural shape and its description. This is a teaching synthesis tool, because it contains multiple specific aspects of an architect’s training. Exploring a physical model allows interaction with its geometry in a direct way and discloses, through tactile and visual exploration, aspects of a shape that normally are only observed (when too far away from the observer/user to be touched).The use of physical models to show the geometries of roofing systems allows seeing the structure from the inside and the outside at the same time. Therefore, we could say that models describe a real object, even if they act as an extreme synthesis (Arnheim 1977).

### Use of Orthographic Projection and Physical Models to Visualize^9^

**Fig. 1:**
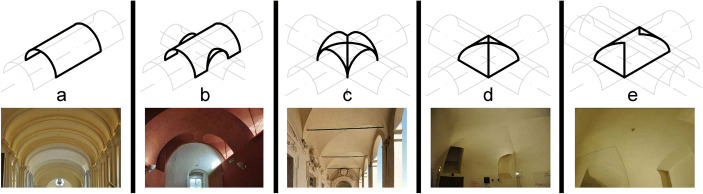
Cylinders intersections generating theoretical vaults, with built examples: a) barrel vault; b) barrel vault with lunettes; c) groin vault; d) cloister vault; e) barrel vault with cloister heads (pictures of the Royal Residence of Venaria Reale in Torino)

To teach roofing/ceiling systems, it is fundamental to build multiple projection planes simultaneously, helping students to construct the three-dimensional model in their mind.The possibility of observing the represented shapes in the architectural environment (looking at real artefacts) becomes an integral part of the lesson.^10^ Working in a heritage context – Valentino Castel, location of the Department of Architecture and Design of the Politecnico di Torino – allows us to make the room(in which we are teaching)itself an object of study. By analysing roofing systems, we can recognize rotation and translation surfaces and we need to choose the most effective models for their investigation. Figure 1 shows some examples of vaults in real architectures. Consequently, the room itself highlights the difficulty of binding the point of view to the intrados (the inner surface) of the ceiling system.

However, a simple exercise in physical modelling allows obviating the problem of the point of view, adding the concept of both scale of representation and symbolic representation (Casale 2017). The physical model is then used to understand geometry through modelling and/or visual/tactile exploration.

### Using Paper Models^11^

We use (and design) paper models to make students discover geometry in a tangible way and to recognize it in every designed manipulation act. The models we use resemble origami models, but some of them are only origami-inspired, because they must also meet specific production requirements. The transformation of the paper sheet becomes, in this situation, an opportunity to pass from meaning to signifier: a metamorphosis in the sense suggested by Deleuze (1988).

An origami model is traditionally obtained from a single sheet of paper, using only folding operations. In fact, a sheet of paper cannot be stretched or sheared, but it can be easily folded, due to paper’s physical properties: thickness and elasticity. So, from a mathematical point of view, an origami model is obtained by a suitable immersion of a sheet of paper (usually a square Ω ⊊ *ℝ*^2^) in three-dimensional space *ℝ*^3^. This immersion can be identified by a map *u*: Ω ⊊ *ℝ*^2^ → *ℝ*^3^, which we may assume is locally one-to-one and isometric. As a consequence, the Gauss curvature of the model *u*(Ω) coincides with the Gauss curvature of Ω, which is zero: therefore, by folding a sheet of paper, only (at least locally) developable surfaces can be obtained. Moreover, *u* is a continuous map, because cutting is not allowed, but, due to the folding, it is not smooth: its singular set is called a crease pattern (CP) by the origamists.

**Fig. 2:**
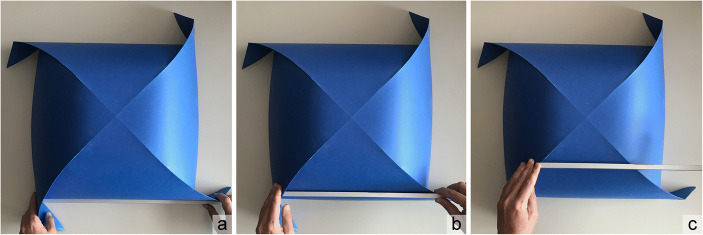
An origami cloister vault. The model is of large format (59 x 59 cm developed and 37 x 37 cm built) to allow visualization in the classroom from the teacher’s desk and it involves a student to check the ruled surface. It is based on a square sheet of paper, on which the CP, that allows the folding, is engraved with a CNC laser engraver

The same mathematical description is the basis of the origami technique to construct ‘rigorously’ models of roofing systems identified by developable surfaces generated by intersections of cylinders. Given cylinder equations, one can obtain the equations of the intersection curves and, referring to arc length calculation, one can describe (by elementary or numerical procedures) the geometric transformations that allows the development of the surface on the plane (Casale et al. 2012; Cumino et al. 2015, 2018). In summary, model use is understood here as a multi-sensory experience, between perception and manipulation, analytical geometric description and design needs. Figure 2 shows the practical sequence proposed in the classroom to verify the belonging of a straight line to a cylindrical surface, by placing a metal rod, normally used as a support for cutting, on it.

**Fig. 3:**
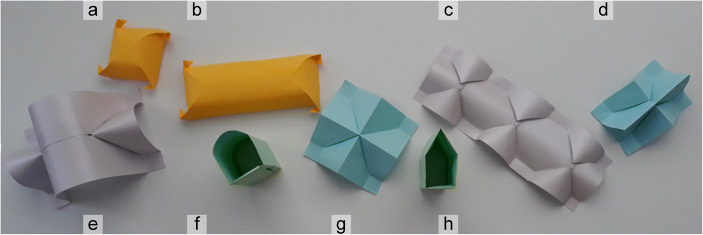
A miscellany of roofing paper models: a) cloister vault; b) barrel vault with cloister heads; c) three groin vaults joined together; d) pitched roof generated by the intersection of two simple roofs with different heights; e) barrel vault with lunettes; f) barrel vault; g) pitched roof; h) barrel vault with linear section – all models, except for f) and h), can be traced back to solid/surface intersections (models made by CC)

Interacting with the model, as a 3D object, its 2D development and the analysis of the curves necessary for its construction became a fundamental part of an educational path and allowed an appreciation of the interdisciplinary approach to the built shape. Starting from ruled surfaces, it is possible to obtain a great variety of origami models, useful for their understanding (see Fig. 3), so that over the years the teaching time dedicated to this topic have been reduced.

**Fig. 4:**
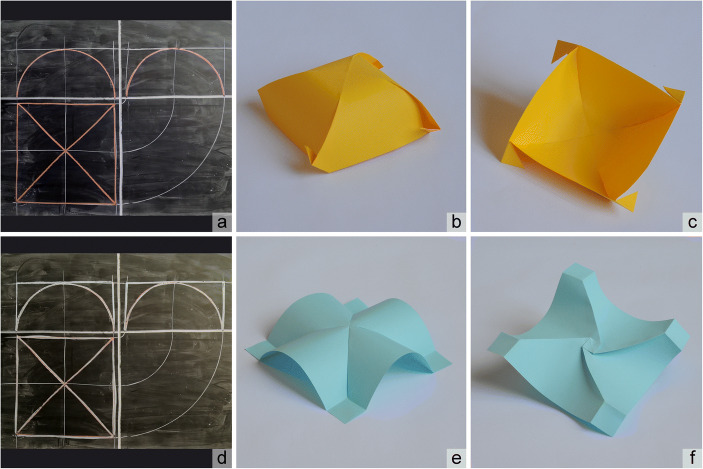
Two vaults generated by intersection of cylinders: a) cloister vault; d) groin vault – the cylinders are the same and it is possible to recognize the shape from thickness and kind of lines (drawings by UZ), while the respective paper models are: b), e) outside view; c), f) inside view

For example, when just drawing (see Fig. 4), the description of cloister and groin vaults forced the teacher to associate an orthographic projection with a 3D view (axonometry or perspective), even though the latter had not yet been explained as a representation tool.

The use of physical models allowed a by-passing of this need by showing the complementarity between these two typologies of vaults. Also, this practice has been of great help for dysgraphia-affected subjects, who have problems with the graphic language: direct observation of a model and/or working on other sensorial spheres makes them able to read the shape without misunderstanding.

## A co-Ordinated Experience between Calculus and the ADSLab: Interdisciplinary Activity on Vaulted Surfaces

**Fig. 5:**
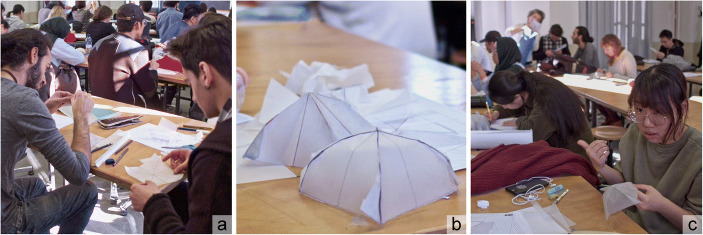
Students at work designing a vault, using graphic tablet and translucid paper (pictures: Pietro Merlo)

At the end of the first semester of the calculus course, we organized an interdisciplinary experience, in order to clarify the relationship between the two disciplines. It drew its origin from other teaching/dissemination tasks and has been described in previous articles (Cumino et al. 2017a; Cumino et al. 2017b; Cumino et al. 2019); thus, here, we report only its main steps. It lasted two hours and dealt with geometric investigations of developable surfaces, conducted with tools that students learned almost distinctly in the two courses, such as orthographic projections and developments, evaluations of areas and perimeters, equations and integral calculus (see Fig. 5).

Held jointly by a mathematician and an architect, the experience made use of unconventional teaching strategies, trying to overcome the traditional isolation of disciplines. It aimed to show mathematics as a tool to describe, evaluate, design and, on the other hand, to show drawing as a tool to visualize and to set up problems mathematically. To this end, problem-solving tasks were proposed, for example to quantify a conservative intervention on simple roofing systems.

**Fig. 6:**
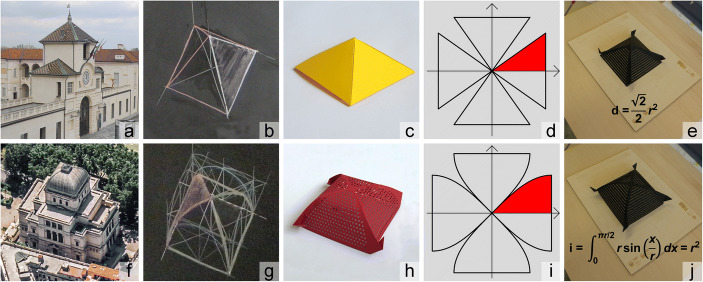
Example of synthesis between mathematical and architectural approaches to study pyramids and cloister vaults: a), f) observation of architectural objects; b), g) graphic reconstruction of such shapes(drawings by UZ); c), h) physical modelling; d) pyramid development; i) cloister vault development, e), j) evaluation by mean of comparison between graphic tablet and physical models of pyramid and cloister vault, with formulas for each respective area

The experience was supported by some blackboard drawing (Fig. 6b, g) and a graphic tablet (Fig. 6e, j), which summarized the geometric description of two surfaces – a pyramid and a cloister vault. Both have identical height and the same orthographic projection; both can be traced back to intersections of cylinders arranged with axes that are orthogonal to each other and belong to the same plane. In the pyramid case, the cylinders have an isosceles triangle as a cross-section; in the cloister vault case, the cylinders have a semi-circular cross-section. The graphic tablet also contains an analytical description of the surfaces, useful to calculate/estimate their areas.

Additional implementations were made during the years 2018–2020, through a better integration of the symbolic/graphic/synthetic descriptions of geometric architectural shapes. As a further step, students were invited to cover the tablet with translucid paper and retrace the eighth part of the vault development to complete it symmetrically. This exercise allowed them to design their personal cloister model and had an impact on geometric understanding of 2D/3D translation of the shape. The hand-made result seemed less precise than the laser-engraved one, but the cognitive process had the efficacy of any learning from engaging in the process.

### Prototyping Physical Models to Share Geometry^12^

As described in earlier sections, we used origami-inspired paper models for studying developable architectural theoretical surfaces (with specific regard to vaulted systems), integrating their use with descriptive and analytic geometry tools. When we started our project, we were obliged to use more ‘hand-craft’ techniques for prototyping models (with common 80, 90 g/m^2^ paper), such as simple paper folding/cutting and die-cut tools (which led us to experiment with 220, 300 g/m^2^ paper, 90 g/m^2^ tracing paper and light acetate sheets). The elaboration of die-cut models forced us to operate on the CP definition, since we needed to design the smallest number of folds that would have been necessary to create our models. Such techniques provided us with very cheap and fast solutions, but their use for even a small ‘mass-production’ was totally inadvisable and unaffordable, at least for the majority of time required.

Within the last year, we were working on the engineering of these models with rapid prototyping tools, in particular a laser engraver. The use of such technology – as well as for the die-cut technique – led to an optimization of modelling in the folding phase. It also made possible a discretization of information type, depending not only on the target model shape, but also on the tool chosen for the CP definition and for the actual possibilities of its realization, even in view of its use by a large audience. In this sense, we were forced to experiment with different types of materials, in order to understand the laser engraver’s strength and weakness points.

We made use of a wide variety of paper supports: first, we tried 150, 200, 220 g/m^2^ paper, also by adding holes and heavy engraving, in order to make the paper more flexible. Then we moved to 160, 300 g/m^2^ paper with cotton fibres, which proved to be the best material for such kind of artefacts. Moreover, the laser engraver led us to produce models with partial abrasion of material, in order to optimize their closure. The appreciation of colleagues who attended our explorations encouraged us in this direction, in order to provide those wishing to reproduce the experience with sets of ready-to-print files that could be rapidly printed with the laser machinery owned by our physical model laboratory.

## Communicate the Geometries of the Vaults: Digital Models to Highlight Perceived Geometries Supporting the Built Shape^13^

To check what students learned through curricular lessons, interaction with physical models (as seen in section before last) and interdisciplinary experiences (as seen in the previous section), we asked them to choose a roofing system generated by the intersection of ruled surfaces among those of the Valentino Castle and to try to communicate it through multiple representation tools experienced during the class. The aim was to obtain homogeneous materials, in order to evaluate critically the impact of experiential teaching with physical models, both through comparison of graphic drawings and through analysis of satisfaction questionnaires.

During the last two academic years, the task was presented as a real competition titled *Communicate Geometries of Vaults* (hereafter, CGV). The poster to be produced was requested to have a strong communicative impact. Moreover, as a minimum requirement, it had to contain framing and location of the chosen vault, a series of photographic images useful for the recognition of basic geometries, their representation in orthographic projection and their 3D digital models. The poster format was mandatory (see below): standards’ definition was indeed a crucial aspect for the subsequent critical evaluation; we proposed them in order to produce heterogeneous posters, thus allowing a great variety of task interpretations.

**Fig. 7:**
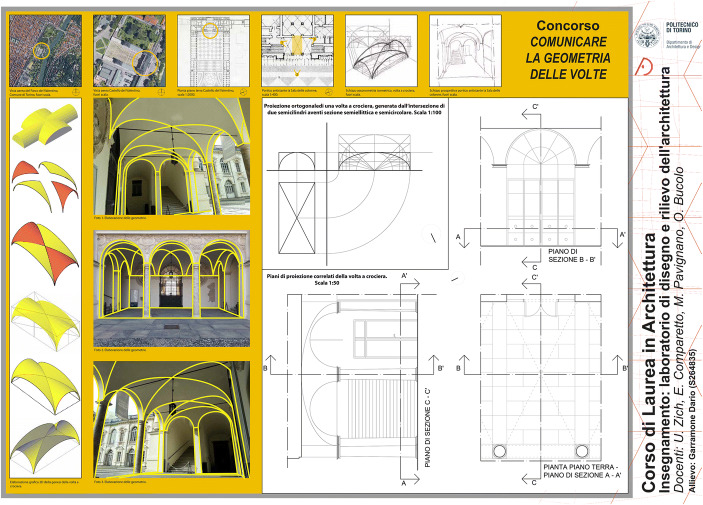
The groin vault – a poster by the student Dario Garramone

In the academic year 2018–2019,^14^ even if the request was explicit – since the vault had to be generated by an intersection of cylinders – only 3.5% of the students chose a sail vault, failing the main competition purpose, while 85% studied a groin vault (Fig. 1c). We present one of the students’ CGV posters in Fig. 7. It has been intentionally inserted without our corrections, in order to be able to observe and discuss critically its potentialities and limits.

Each student had to produce a poster including: a contextualization of the object of study through a series of cartographic representations at different scales, an orthographic projection according to the graphic conventions of the architectural drawing, a simplified scheme to clarify hidden geometries of the studied vault, some photographs and/or sketches illustrating the recognition of the such geometries, one or more 3D models showing how the interaction of hidden geometries generated the vault. We considered quality and quantity of information each student deemed useful to describe the geometry of the studied vault as evaluation parameters: i.e. 3D models’ level of detail, choice of views to show the intrados(inside surface) and/or the extrados (outside surface), transposition of 3D models into images to be included in the poster.

The producer of the poster in Fig. 7 was able to recognize the correct hidden geometries of the vault (two intersecting right hemicylinders, the red one with a semi-circular directrix and the yellow with an elliptical one) and he elaborated coherent 2D and 3D representations. On the other hand, his graphic elaborations of photographic images were not so effective, because he did not use the same colours as the 3D models. Figure 7 also highlights a good balance between hand-made sketches and digital drawings.

**Fig. 8:**
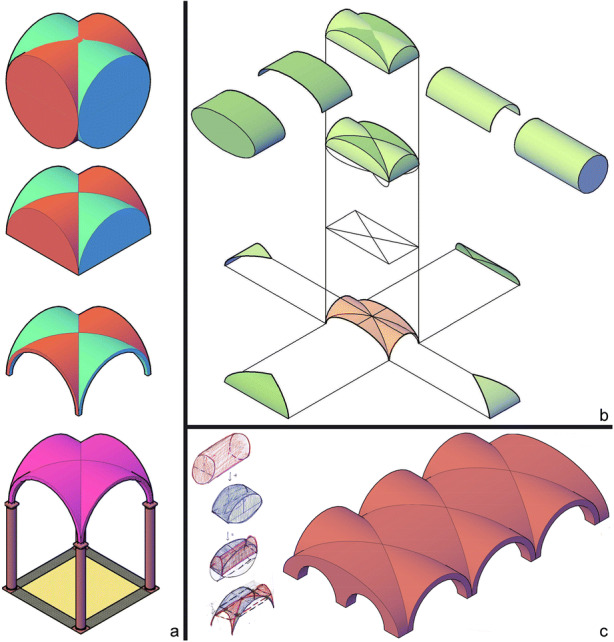
Examples of students’ digital study of generation of groin vaults: a) Issam Fannane; b) Adriana Lauretta; c) Cecilia Egidi

Digital models presented in Fig. 8 highlight different ways of reading and interpreting architectural shapes and even different interpretations of hidden geometries. For example, Figs. 8a and 8b expose the geometric genesis as a sequence of constructive steps of the digital model. The first one is the simplest, because the student did not highlight intersections curves between the two cylinders, while the second presents a more critical analysis of the geometric genesis of the related surface. The model shown in Fig. 8c must be integrated with sketches, in order to understand how the student interpreted the vault, since she modelled the entire vaulted system and not just a single element. Nevertheless, students’ models in general show how graphic representations of a spatial/geometric problem solving are subject to each student’s personal interpretation.

## The Impact of Physical Models from Students’ Points of View^15^

The reasons that led our teaching actions to be strongly characterized by the use of models are various and closely related to our disciplinary specificity. However, new questions arose from the interdisciplinary synergy on how to evaluate the impact of these proposals within the training course: are these actions a teacher’s optional choice or do we consider them so foundational as to be proposed as part of the architectural studies curriculum? How much geometry is needed for and how to teach it to young architecture students?

In the past few years, we have introduced innovations and experiments separately into our respective teaching of calculus and ADSLab lessons. In response to the hypothesis of sharing them with the entire degree course, we have had to systematize our proposals. Therefore, it was necessary to go beyond the teacher’s point of view and build assessment tools that took the students’ perceptions of engaging these tasks into account. In 2019, the students’ point of view was investigated through a series of questions proposed in the form of a satisfaction questionnaire.

The questionnaire was organized in four blocks which collected participants’ general characteristics and the use of the models during the various tasks. Students were asked to give an evaluation; the answers had a six-level scale: 1 ‘Very bad’ to 5 ‘Excellent’ and 6 ‘I do not know’. In the questionnaire, we analysed how students perceived the usefulness of physical models for shape visualization and understanding, by assessing whether the models played a role in the formation of their visual–spatial abilities and the relation between mathematics topics and drawing actions. The 39 student responses settled decisively in around 4.5. As for the usefulness of mathematical topics for active participation, 56% of students gave marks from 4 to 5, with the median and mode mark of 4 and the arithmetic mean of 3.5. About the usefulness of this experience to understand mathematical topics inherent in architectural shapes, we obtained the same percentage and statistic indices.

This leads to a reflection: mathematics teaching proposals can be more effective for students if referred to architectural examples. In particular, the use of models during the calculus lessons could allow students to move from the concrete to the abstract in the learning process of mathematics.

Table 1 summarizes these results, without including the few students who responded with 6 s in the scale. It also allows us to deduce that the arithmetic mean, median and mode were always between 4 and 5.

**Table 1:** Extracted results collected by the satisfaction questionnaire.

**Investigated topics. Usefulness of:**	A. mean	Mode	Median
*Physical models to visualize architectural shapes*	4.5	5	5
*This experience to understand architectural shapes*	4.5	5	5
*This experience to improve visuo-spatial abilities*	4.4	4	4
*This experience to improve graphic communication abilities of geometric genesis processes*	4.2	5	4
*This experience to improve digital modeling skills*	4.4	4	4
*This experience to understand related mathematical topics*	3.6	4	4
*Mathematical topics to participate actively*	3.5	4	4

## Conclusions

We presented a set of interdisciplinary teaching experiences between mathematics and drawing for first year Architecture undergraduate students, focused on some common issues. In planning those tasks, our main goal was to foster students’spatial skills and comprehension of applicational interconnections between architecture and mathematics, using geometry as a shared common language, and proposing paper models as tools for understanding the geometric construction of architectural shapes.

Among the most recent research on physical models for architecture – especially for some interdisciplinary issues – an important role is played by the use of paper-based physical models, inspired by three-dimensional origami models, which have low cost and specific dynamic qualities.

Origami-inspired paper models are an excellent tool for visualizing ruled surfaces and for understanding their intersections. However, their use should be highly structured, because tactile exploration of origami models can occasionally lead to errors of interpretation or not be sufficient to read the geometry in its complexity. In this sense, we should not forget that the scaled/tangible artefact has assumed the role of simple physical representation, directly explorable by visual means (Pavignano et al. 2020). Indeed, what seems really important to deepen conceptual understanding is to promote students’ abilities in connecting different representations: visual and symbolic, formal and informal, analytic and perceptual, rigorous and intuitive (Noss et al. 1997).

We have verified over the years the effectiveness of paper models in speeding up the teaching of different types of compound vaults and in conveying the underlying mathematics, without compromising its quality. Subsequently, we have applied this experience to other tasks: for example, evaluation of vaulted surfaces has already had its evolution from the first test up to the present day, in getting to the model design. Also, in our experience, the use of digital tools revealed high potential, not only as a means of understanding architectural shapes, but also as a production instrument for physical models in the use of a laser engraver to facilitate paper-folding. As we got good feedback both from students and from colleagues, we feel the need for further research to overcome the compartmentalized way of students’ thinking and to promote their flexibility in moving between different registers of representation (the architectural and the mathematical).

## Footnotes

1 Even Mario Salvadori, while remembering his years as an engineering student, wrote: “I began first to fear and then to hate math” (cited in Williams and Nastasi 2007, p. 166).

2 From the description of our calculus course, available on-line: www.polito.it.

3 Written by MP and UZ.

4 Original text: “l’Architettura, come facoltà, che in ogni sua operazione adopera le misure, dipende dalla Geometria, e [l’Architetto] vuol sapere almeno i primi suoi elementi […]”.

5 Written by CC and MP.

6 Written by MP.

7 Written by CC.

8 Written by UZ.

9 Written by UZ.

10 This teaching approach was experimented not only in university courses, but also at other school levels within an interdisciplinary research project which involved two Departments of Politecnico di Torino (DAD and DISMA) and the Centro Studi Residenze Reali Sabaude (www.lavenaria.it/en/research-center). This project promoted guided tours, introducing geometry – in a cultural heritage context – as a tool for understanding architectural shapes (Armand et al. 2018).

11 Written by CC and UZ.

12 Written by CC, MP and UZ.

13 Written by MP and UZ.

14 For the academic year 2019–2020, we had planned to double the task by having them produce two posters, the first one more directed to the digital modelling (during the first semester) and the second one more focused on the built survey (during the second semester). Due to the COVID-19 pandemic, however, we were not able to complete this second task.

15 Written by MLS.
